# Fluorescence dynamics of the biosynthesized CdSe quantum dots in *Candida utilis*

**DOI:** 10.1038/s41598-017-02221-1

**Published:** 2017-05-17

**Authors:** Li-Jiao Tian, Nan-Qing Zhou, Xian-Wei Liu, Xing Zhang, Ting-Ting Zhu, Ling-Li Li, Wen-Wei Li, Han-Qing Yu

**Affiliations:** 1 Department of Chemistry, University of Science and Technology of China, Hefei, 230026 China; 2 School of life Sciences, University of Science and Technology of China, Hefei, 230026 China

## Abstract

Organisms served as factories of bio-assembly of nanoparticles attracted a lot of attentions due to the safe, economic and environmental-benignity traits, especially the fabrication of the super fluorescence properties quantum dots (QDs). However, information about the developmental dynamics of QDs in living organisms is still lacking. In this work, we synthesized cadmium-selenium (CdSe) QDs in *Candida utilis* WSH02-08, and then tracked and quantitatively characterized the developmental dynamics (photoactivation, photostable and photobleaching processes) of bio-QDs by translating fluorescence microscopy movies into visual quantitative curve. These findings shed light on the fluorescence properties of the bio-assembled QDs and are expected to accelerate the applications of the synthesized QDs *in vivo*. It provided a new way to screen bio-QDs and monitor the quality of QDs *in vivo*.

## Introduction

Quantum dots (QDs) are nano-scale fluorescent materials with high photo-stability, narrow and size-tunable emission spectra, and broad spectral window^1^. These amazing optical properties of QDs have motivated intensive research interests from material scientists, chemists and biologists worldwide to explore their massive production and potential applications^2–4^. Biosynthesis, with many advantages such as facile operation, low cost and environmental benignity, is considered as a promising approach to realize large-scale production of QDs^5, 6^.

To date, an increasing number of *in vivo* biosynthesized QDs (bio-QDs) with excellent hydrophilicity and inherent biocompatibility by various types of microorganisms, from single-cellular bacteria^7–9^ to higher-level animals^10^, have been reported. For the practical bioimaging/biomedical applications of bio-QDs, several aspects of fluorescence properties are of particular interests: fluorescence intensity; sensitivity to photoactivation (i.e., the rate of fluorescence enhancement under light excitation)^11, 12^; and photostablility^13^. High photostablility means a slow photobleaching (the loss of fluorescence under prolonged illumination) and a long photostable lifetime (here defined as the time span when the fluorescence intensity is no less than 90% of their peak value)^14, 15^. To evaluate the fluorescence properties, it is usually to extract the QDs from biomass carefully and remove the proteins from bio-QDs, which is time-consuming and not friendly to screen bio-QDs with high quality in industrial production^16, 17^. Therefore it is of significance to study the fluorescence properties of bio-QDs *in vivo*, especially the developmental dynamics of bio-QDs in living organisms.

In this work, we applied *Candida utilis* WSH02-08^18^, to synthesize cadmium-selenium (CdSe) QDs, and probed and tracked the developmental dynamics of *in vivo* synthesized CdSe bio-QDs in living cells. The fluorescence intensity, photoactivation rate and index, photostable lifetime, and photobleaching decay time of the obtained CdSe bio-QDs were evaluated. To facilitate real-time monitoring of the fluorescence emission dynamics of the bio-QDs *in vivo*, we extracted their fluorescence evolution by translating fluorescence video into visual curves as a monitoring tool. This study could enable a better understanding of the fluorescence properties of bio-QDs. It also will provide a new way to screen bio-QDs and monitor the quality of QDs *in vivo*. This quick feedback may be beneficial for optimizing the parameters for the large-scale biosynthesis of QDs.

## Results and Discussion

### Bio-assemble of QDs in *Candida utilis* WSH02-08

**Figure 1:**
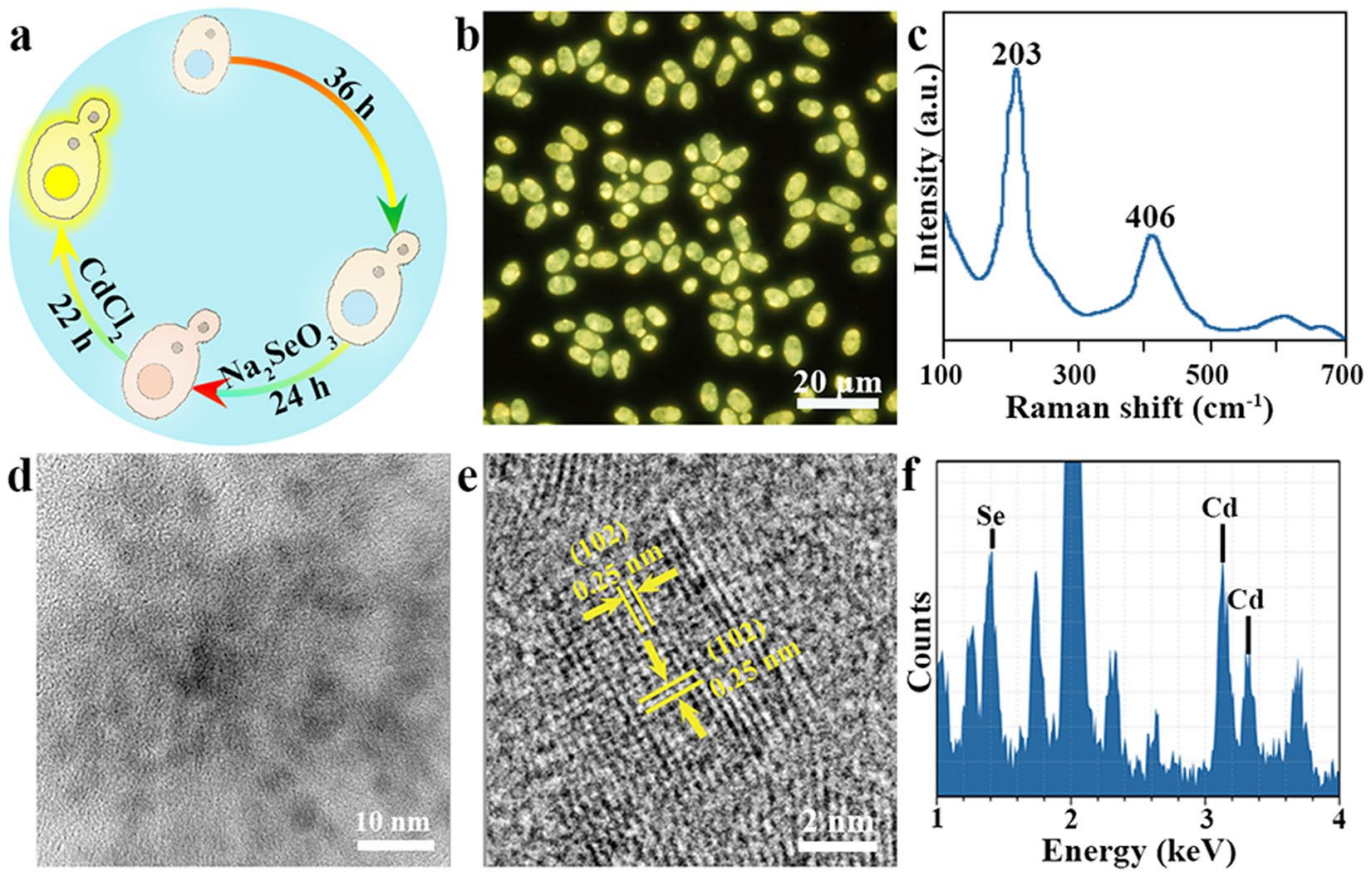
Characteristics of the CdSe QDs synthesized by *C*. *utilis* WSH02-08 with 1 mM Na_2_SeO_3_ and 6 mM CdCl_2_. (**a**) Schematic illustration of bio-QDs fabrication process by *Candida utilis* WSH02-08. (**b**) Fluorescence microscopy image of cells exposed to Cd and Se salts. (**c**) *In situ* micro-Raman spectrum of the cells. (**d**) High-resolution transmission electron microscopy (HRTEM) image of the purified QDs. (**e**) The lattice planes spacing of the purified QDs is 0.25 nm. (**f**) X-ray energy-dispersive spectroscopy (EDS) analysis of the purified QDs.

In order to synthesize CdSe bio-QDs, the *C*. *utilis* WSH 02-08 was cultivated with 1 mM sodium selenite (Na_2_SeO_3_) and 6 mM cadmium chloride (CdCl_2_) (Fig. 1a). At the end of the synthesis process, the cells exhibited bright yellow fluorescence observed by fluorescence microscopy (Fig. 1b). The characteristic peaks of CdSe (203 cm^−1^ and 406 cm^−1^ assigned to longitudinal optical (LO) and 2LO phonon of Cd-Se, respectively) were illustrated by *in situ* Raman spectrum. In order to get more detail morphology and structure information of the QDs, the nanoparticles were purified and analyzed by high-resolution transmission electron microscopy (HRTEM) and X-ray energy-dispersive spectroscopy (EDS). The purified QDs yield strong Se, Cd signals and the average diameter is 4.38 ± 1.30 nm (Fig. 1d and f). The HRTEM shows continuous lattice fringes with an interplanar lattice spacing of 0.25 nm, corresponding to the (102) plane of CdSe (Fig. 1e). All these evidences confirm the synthesis of CdSe QDs inside the cells of *C*. *utilis* WSH02-08.

### Fluorescence dynamics of the bio-QDs

**Figure 2:**
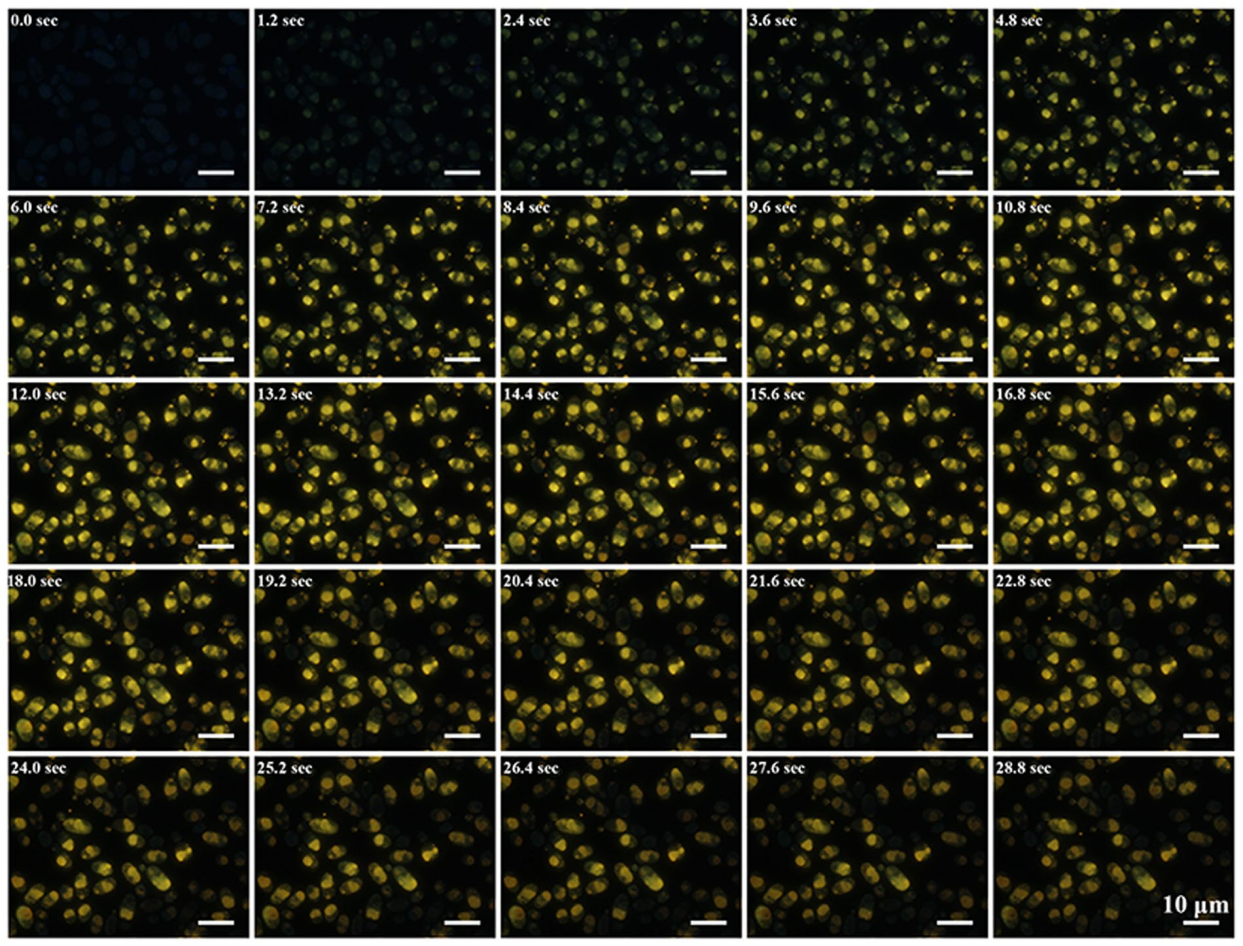
Time series fluorescence microscopy images of the cells to show the fluorescence evolution of the bio-QDs *in vivo*. The cells were grown in medium with 1 mM Na_2_SeO_3_ and 2 mM CdCl_2_. The time interval between two images was 1.2 second. Video was taken from a 150-μm^2^ area where there were at least 80 cells.

To examine the fluorescence properties of the CdSe QDs, we performed *in vivo* long term recording of fluorescence dynamics of the bio-QDs using fluorescence microscope. Interestingly, the fluorescence intensity of the bio-QDs in *C*. *utilis* WSH02-08 declined after 30 min illumination, suggesting a photobleaching process. To short the recording time, the bio-QDs synthesized at a lower Cd precursor concentration (i.e., 1 mM Na_2_SeO_3_ and 2 mM CdCl_2_) were selected here for visualization of the fluorescence evolution over time (Fig. 2). Dim fluorescence appeared at 1.8 seconds after the illustration and kept strengthening till reaching a plateau. After about 13.8 seconds, the fluorescence started to decrease.

**Figure 3:**
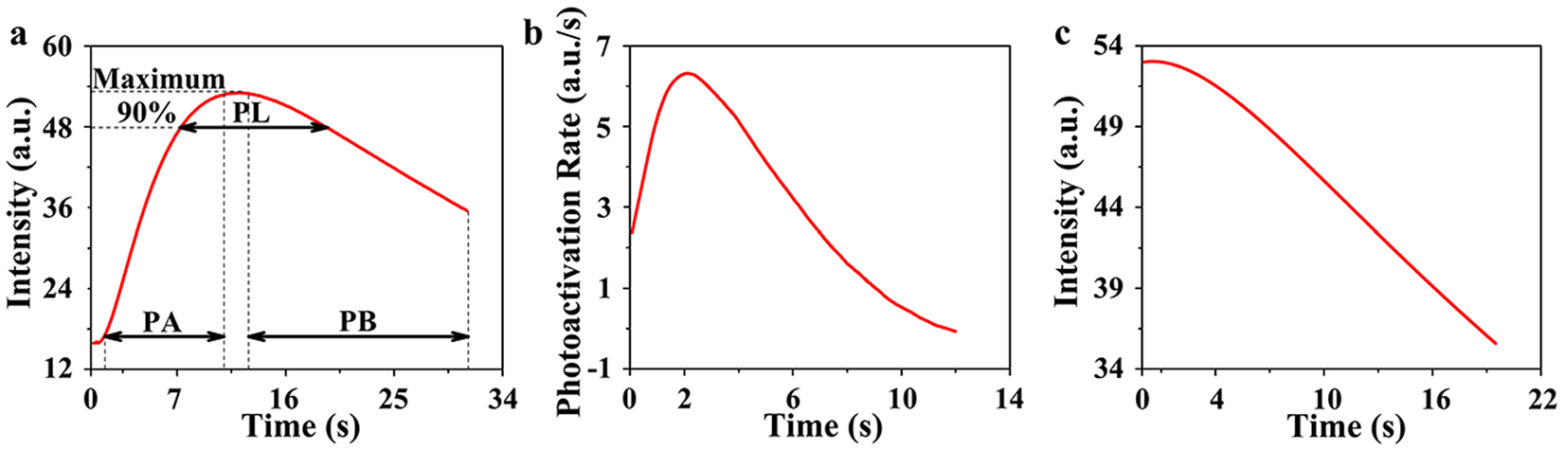
Fluorescence curves of the bio-QDs in cells over time. (**a**) Variation of intracellular mean fluorescence intensity that shows the processes of the photoactivation and photobleaching, and photostable lifetime; (**b**) The derivative of non-linear function of the photoactivation process reveals the photoactivation rate; (**c**) Best-fit bi-exponential form of the photobleaching curve. PA, PL and PB represent photoactivation, photostable and photobleaching process, respectively.

**Figure 4:**
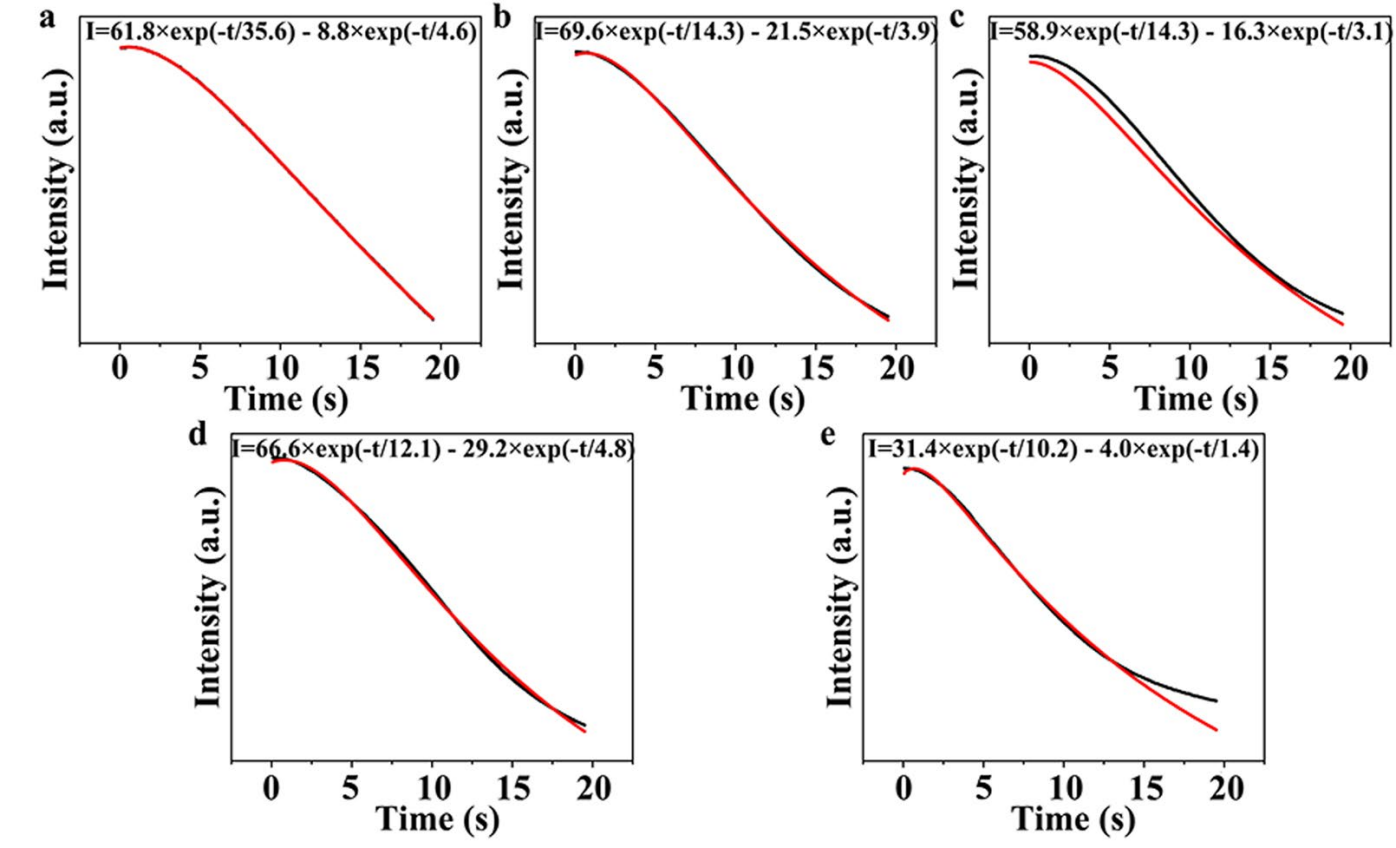
Photobleaching process exposed to different selenium contents, and the bi-exponentials (*I* = *a*_*1*_exp[−t/*τ*_*1*_] + *a*_*2*_exp[−t/*τ*_*2*_]) fitted line. The red line represents the fitted data and the dark line represents experiment values. (**a**) Se1Cd2, (**b**) Se2Cd2, (**c**) Se3Cd2, (**d**) Se4Cd2, (**e**) Se5Cd2.

To quantitatively describe the fluorescence dynamic changes of the bio-QDs synthesized in *C*. *utilis* WSH02-08, we plotted the average fluorescence intensity of the whole image over time using image J software^19^. Four parameters of the fluorescence dynamics, i.e., the photoactication index and photoactivation rate, photostable lifetime and photobleaching decay time, were evaluated. The photostable lifetime of the bio-QDs was 12.4 seconds (Fig. 3a), while the photoactivation rate increased initially to 6.314 within 3 seconds and then decreased (Fig. 3b). Photoactivation process fit well with the stretched-exponential law (I = I_0_ − Aexp − [t/τ]^β^). In this function, I is the fluorescence intensity, I_0_ is the highest value of the fluorescence intensity, τ is the photoactivation half time, in which the fluorescence intensity reaches the value of I_0_/2. A is a scaling coefficient, we called photoactivation index here and β is a stretching parameter. The best-fit results are presented in Fig. S1 and Table S1. The photobleaching process of the bio-QDs exhibites exponential decay rule and the photobleaching data were well fitted by the bi-exponential form of “a_1_exp(−t/τ_1_) + a_2_exp(−t/τ_2_)”, where *τ*_*1*_ represents the fast decay and *τ*_*2*_ represents slow decay, and *a*_*1*_ and *a*_*2*_ are the amplitudes of the exponentials^20^. The photobleaching consisted of fast decay and slow decay (Fig. 3c). The estimated fast decay time (the dominant parameter to judge the bleaching rate) was 35.57 seconds (Fig. 4a).

### Tuning the fluorescence properties of the bio-QDs

The fluorescence properties of the above bio-QDs synthesized by *C*. *utilis* WSH02-08 at different CdCl_2_ concentrations varied substantially, suggesting a considerable impact of cultivation conditions on the bio-QDs synthesis process and a feasibility of regulating the bio-QDs properties by adjusting the precursor concentration.

**Figure 5:**
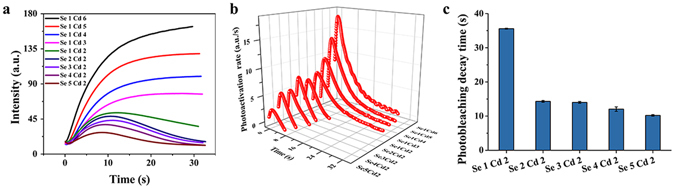
Fluorescence properties of the bio-QDs in *C*. *utilis* WSH02-08. (**a**) Intracellular mean fluorescence intensity trajectories in different synthesized bio-QDs recorded under lamp illumination using fluorescence microscopy; (**b**) The derivative of non-linear function of the photoactivation process under different synthesis conditions; (**c**) Best-fit photobleaching decay time constants for the bi-exponential form of the bio-QDs. All the experiments were conducted under the same conditions.

To validate this hypothesis, we comparatively evaluated the photoactivation index and rates, photostable lifetimes and photobleaching decay times of the bio-QDs in the WSH02-08, which were synthesized at 9 different combinations of precursor concentrations. As expected, substantial differences in the fluorescence intensities and dynamics of the bio-QDs were observed for the 9 tested groups (Fig. 5a). The Se1Cd6 group exhibited the maximum fluorescence intensity (162.6), while the Se5Cd2 group showed the minimum (28.5). In addition, the Se1Cd6 group also had the longest photostable lifetime, the fastest photoactivation rate and the maximum photoactivation index (Fig. 5b and S1). Here, Se1Cd6 represents the precursor concentration is 1 mM Na_2_SeO_3_ and 6 mM CdCl_2_, while Se5Cd2 represents the precursor concentration is 5 mM Na_2_SeO_3_ and 2 mM CdCl_2_. From the fitted curves, the average values of *τ*_1_ under different Se content synthesis conditions are summarized in Fig. 4. The fastest decay time was observed for the Se5Cd2 group, indicating that a higher Se content could accelerate the photobleaching of the bio-QDs in WSH02-08. These results indicate that a higher Cd and a lower Se content would increase the photoactivation rate and photostable time of the bio-QDs, and decrease the photobleacing rate, resulting in better photostable bio-QDs. These results also reflect that such fluorescence dynamic parameters are facile index to screen good fluorescence properties QDs.

## Conclusion

In summary, we synthesized bio-QDs using *C*. *utilis* WSH02-08 and monitored their fluorescence dynamics *in vivo*. The critical parameters to evaluate the bio-QDs, e.g., fluorescence intensity, photostable lifetime, photoactivation and photobleaching decay time, were obtained and quantified. In addition, higher Cd contents are found to favor the formation of the bio-QDs with a higher fluorescence intensity and photoactivation index in *C*. *utilis* WSH02-08, implying the possibility of using fluorescence dynamics parameters as screen index. Benefited from these results, the preparation of bio-QDs with various compositions (e.g., CdTe, CdS) by different microorganisms can be expected, which may ultimately make the bioimaging/biosensing applications of bio-QDs a real possibility. Therefore, our work provides an opportunity to gain new microscopic insights into the developmental dynamics of bio-QDs and their fluorescence properties in microbial cells, and is expected to accelerate the pace of development of environmentally friendly and cost-effective ways for bio-assembled QDs.

## Methods

### Biosynthesis of CdSe QDs in *Candida utilis*

*Candida utilis* WSH02-08 strain was cultured into the medium (30 g/L glucose, 10 g/L ammonium sulfate, 6 g/L yeast extract, 4.5 g/L potassium dihydrogen phosphate, 0.75 g/L magnesium sulfate, 14.7 g/L sodium citrate dehydrate and pH is 5.5) for 36 h at 30 °C under 200 rpm sharking in aerobic condition. The activated strain was incubated with Na_2_SeO_3_ and glucose (10 g/L) for another 24 h. Then CdCl_2_ was added to finish the QDs fabrication for another 22 h. All the bio-assembled QDs process were conducted at 30 °C under 200 rpm sharking in aerobic conditions. After the synthesis procedure, we collected the cells by centrifugation (6000 g, 4 °C, 5 min) and washed two times with 10 mM Tris-Cl (pH = 7.6).

### Fluorescence microscopic observation

To monitor the fluorescence dynamics process of the bio-QDs *in vivo*, the collected cells were observed using a wide field fluorescence microscope (BX-51, Olympus Co., Japan) with a wideband MWU2 filter (Ex 330–385 nm). All images were taken using a water immersion objective (100×) under 120 W mercury lamp (X-Cite 120 Q) irradiation. Furthermore, the images and videos were recorded by the DP2-BSW software (Olympus Co., Japan) under identical conditions. The videos were recorded at 15 frame/s with a CCD camera (DP72, Olympus Co., Japan) and the recording duration is limited to 1 minute in order to make sure the viability of the cells.

### Raman measurements

*In situ* Raman spectrum was obtained using a Thermo Scientific^TM^ DXR^TM^xi spectrometer. The excitation wavelength was 532 nm and using the 100× objective to record. Silicon wafer was used to automatically calibrate the instrument wavelength and the samples were placed on a quartz plate.

### Characterization of the purified QDs

The synthesized bio-QDs were collected and resuspended in 10 mM Tris-Cl (pH = 7.6). To isolate the QDs, the resuspended cells were disrupted by high pressure cell disruption device. The crushed cells were then sonication (2 s with 5 s intervals) for 20 times in ice bath. The suspension was centrifuged at 4 000 g for 10 min to collect the fluorescent supernatant. The resulting supernatant was concentrated and washed using a 50 kDa tubular ultrafiltration membrane (MWCO-10000, Merck Millipore Co., USA). To digest the protein impurity, 100 μg/mL proteinase K was added and treated at 37 °C for 1 h. Then resulting solution was purified by centrifugation (15000× g, 10 min) and washing by 50 kDa tubular ultrafiltration membrane and dialysis. Finally, the obtained purified QDs were subjected to high-resolution transmission electron microscopy analysis (TEM-JEM-2010F, JEOL, Tokyo, Japan).

## Electronic supplementary material

Supporting Information
