# Supplementary material for: Fluorescence dynamics of the biosynthesized CdSe quantum dots in Candida utilis
Source: Sci Rep. 2017 May 17;7:2048. doi: 10.1038/s41598-017-02221-1 (PMC5435690; doi:10.1038/s41598-017-02221-1)
Supplement: Supplementary file 1 — Supporting Information [file 41598_2017_2221_MOESM1_ESM.pdf]

Supporting Information

Fluorescence dynamics of the biosynthesized CdSe quantum dots in *Candida utilis*

Li-Jiao Tian[1], Nan-Qing Zhou[1], Xian-Wei Liu[1,*], Xing Zhang[1], Ting-Ting Zhu[4], Ling-Li Li[1], Wen-Wei Li[1,*], Han-Qing Yu[1]

[1]Department of Chemistry, [4]School of life Sciences, University of Science and Technology of China, Hefei, 230026, China

***Corresponding author:**

Prof. Wen-Wei Li, Fax: +86 551 63601592; E-mail: wwli@ustc.edu.cn

Prof. Xian-Wei Liu, Fax: +86 551 63601592, E-mail: xianweiliu@ustc.edu.cn

Table S1. Best-fit photoactivation parameters for stretched exponentials form of in vivo synthesis of CdSe

	I_0	A	τ (s)	β
se1cd2	53.80	-37.67	4.48	1.52
se1cd3	77.67	-65.71	6.99	1.31
se1cd4	99.29	-90.62	7.19	1.19
se1cd5	128.51	-118.85	6.96	1.18
se1cd6	162.61	-155.31	7.29	1.00
se2cd2	49.79	-36.54	3.80	1.41
se3cd2	44.38	-30.33	4.24	1.40
se4cd2	38.57	-25.14	3.49	1.55
se5cd2	28.49	-14.55	3.73	1.58

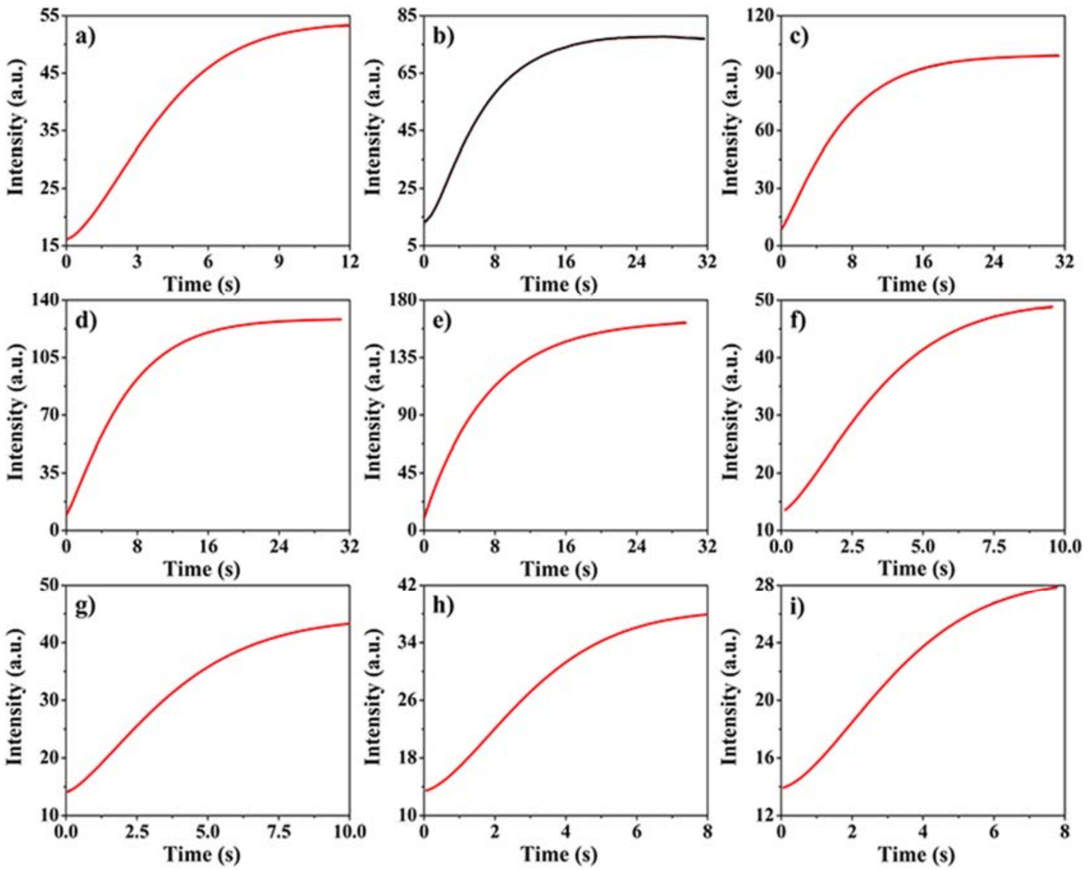

Figure S1. Photoactivation behavior exposed to different metal content ,and the stretched exponentials($I = I_0 + A\exp[-t/\tau]^{\beta}$) fitted line. The red line is fitted data and the dark line is experiment values. a: Se1Cd2, b:Se1Cd3, c:Se1Cd4, d:Se1Cd5, e:Se1Cd6, f:Se2Cd2, g:Se3Cd2, h:Se4Cd2, i:Se5Cd2 .
